# A Case of an Infantile Lingual Leiomyomatous Hamartoma

**DOI:** 10.1155/2022/5377771

**Published:** 2022-10-11

**Authors:** Thomas Z. Gao, Austin Schafer, Abdulrahman Althubaiti, Bonita Fung, Charles Elmaraghy

**Affiliations:** ^1^The Ohio State University Wexner Medical Center, 410 W 10th Ave, Columbus, OH 43210, USA; ^2^Nationwide Children's Hospital, Department of Otolaryngology, 700 Children's Dr, Columbus, OH 43205, USA

## Abstract

Lingual leiomyomatous hamartomas are rare lesions of the tongue with largely unknown mechanisms of formation. These lesions are often asymptomatic, though they may present with symptoms, particularly relating to swallow function. Workup should include imaging of the head and neck, and diagnosis should be made histologically. Treatment is surgical excision. This case is a report of a 4-week-old female who presented for evaluation of an asymptomatic 1 × 1 cm dorsal midline tongue mass discovered at birth. The patient was monitored until the age of 9 months, at which time the mass was surgically excised. The patient had an uncomplicated postoperative course. Pathological analysis yielded a diagnosis of leiomyomatous hamartoma.

## 1. Introduction

Lingual leiomyomatous hamartomas are rare, often asymptomatic lesions that are defined by the predominance of smooth muscle on histological examination [1]. In the pediatric population, lingual hamartomas make up approximately 13% of tongue lesions and typically follow cystic, polypoid, vascular, and neoplastic lesions in the differential diagnosis [2–4]. Per our literature review, there have been 29 reported cases of lingual leiomyomatous hamartomas, which reportedly occur predominantly on the dorsal tongue and tongue base [5–25]. In this present case, we describe the evaluation and subsequent treatment of an asymptomatic dorsal midline lingual leiomyomatous hamartoma in a 4-week-old female.

## 2. Case Presentation

**Figure 1:**
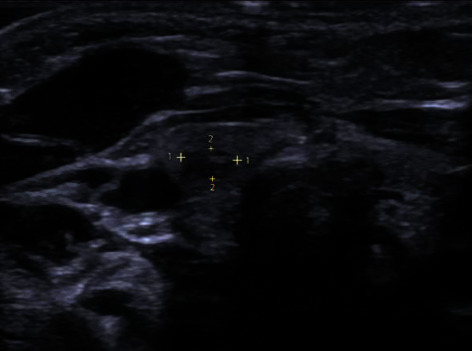
Ultrasound of the neck demonstrating 3.7 × 2.0 × 3.1 mm nodule in the right thyroid lobe.

**Figure 2:**
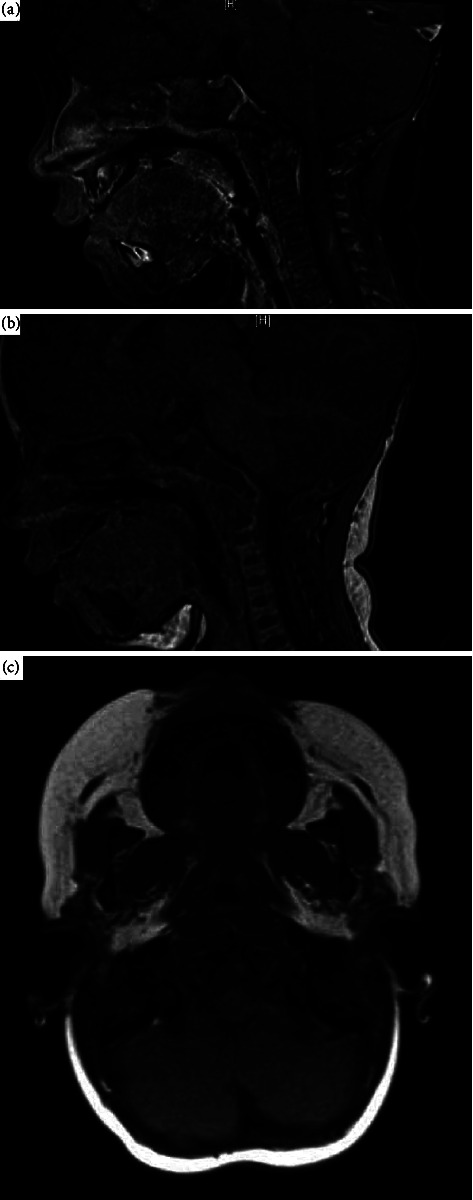
Magnetic resonance imaging (T1 with contrast, (a) 3D MPRAGE, (b) T1 without contrast), and (c) of the head demonstrating a polypoid dorsal midline lingual mass.

An otherwise healthy 4-week-old female born at 34 weeks without complications presented to our otolaryngology clinic with an asymptomatic, stable tongue mass originally discovered at birth. On exam, a firm, nonfriable, 1 × 1 cm lesion was appreciated at the level of the dorsal midtongue, anterior to the foramen cecum. No further abnormalities were noted. It was recommended that the lesion be excised between the ages of 6–12 months. After multiple surveillance visits, the decision was made to surgically excise the lesion when the patient was 9 months old. Prior to the operation, an ultrasound of the neck was obtained to confirm the presence of a thyroid gland, given the known tongue lesion. The thyroid was present; however, the ultrasound demonstrated a 3.7 × 2.0 × 3.1 mm nodule in the right lobe, which was interpreted as likely intrathyroidal ectopic thymic tissue (Figure 1). A follow-up MRI did not capture this nodule. However, the tongue lesion was visualized (Figure 2).

### 2.1. Operation

A 3.0 silk suture was placed down the midline of the tongue for retraction, and the lesion was completely excised with 1 mm margins using a guarded needle tip Bovie. The mucosa was then reapproximated with a 4.0 vicryl suture. The mass was sent for pathological analysis as a fresh specimen. Blood loss was minimal and the patient had no postoperative complications.

### 2.2. Pathological Findings

**Figure 3:**
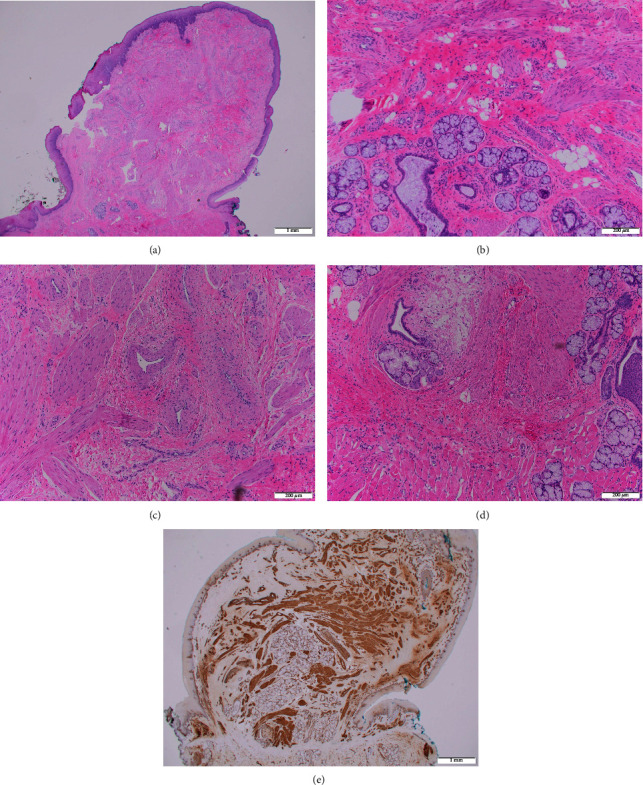
(a) An histological section of lingual mass demonstrating a raised, broad-based polypoid lesion covered with stratified keratinizing epithelium with a central core of smooth muscle (hematoxylin and eosin stain, 2x original magnification). (b) An histological section of lingual mass demonstrating smooth muscle with the salivary gland and duct tissue with intervening adipocyte clusters (hematoxylin and eosin stain, 10x original magnification). (c) An histological section of lingual mass demonstrating smooth muscle with small, thick-walled vessels present (hematoxylin and eosin stain, 10x original magnification). (d) An histological section of lingual mass demonstrating transition between smooth muscle of leiomyomatous lesion and skeletal muscle of dorsal tongue (hematoxylin and eosin stain, 10x original magnification). (e) An histological section of lingual mass with smooth muscle actin stain (2x original magnification).

Gross examination of the specimen demonstrated a tan-white, semi-firm, glistening nodule, measuring 0.9 × 0.7 × 0.5 cm. Histological analysis revealed a raised, broad-based polypoid lesion covered with stratified keratinizing epithelium with a central core of smooth muscle (Figure 3(a)). Smooth muscle was visualized with small, thick-walled vessels, and mucinous salivary gland and ductal tissue, with clusters of adipocytes present within the intervening stroma, all adjacent to native lingual skeletal muscles (Figures 3(b)–3(d)). The smooth muscle actin staining was performed to further clarify the smooth muscle present in the lesion (Figure 3(e)). These findings were most consistent with the diagnosis of a leiomyomatous hamartoma.

## 3. Discussion

Hamartomas are heterogeneous lesions made of disorganized native tissue that may occur in a variety of locations throughout the body, and these lesions are defined by their predominant tissue type [1]. Lingual hamartomas are relatively uncommon lesions of the tongue, making up approximately 13% of primary pediatric tongue lesions [2]. The differential for a pediatric tongue lesion is broad, encompassing macroglossia, polyps, cysts (e.g., enteric duplication cysts, thyroglossal duct cysts, and vallecular cysts), vascular lesions (e.g., arteriovenous malformation and lymphatic malformation), or neoplasia (e.g., sarcoma, lymphoma, and schwannoma) [3, 4]. Per our literature review to date, there are a total of 28 cases of lingual leiomyomatous hamartomas, or smooth muscle-predominant hamartomas, reported in the literature, with 19 of them being on the dorsum of the tongue, as in our present case (Table 1) [5–25]. Other locations of these hamartomatous lesions in the oral cavity include the anterior maxilla and the hard palate [24, 27].

Many patients with lingual hamartomas will present asymptomatically. In symptomatic children, feeding difficulties seem to predominate, including recurrent emesis, swallowing dysfunction, and failure to thrive and less commonly, signs of airway obstruction (e.g., snoring) [22, 26]. Clinical characteristics supportive of a lingual hamartoma include, but are not limited to, a small lesion (less than 1.5 cm in largest diameter), a pink, pedunculated appearance, and a midline location on the tongue [22]. The mechanism of lingual hamartoma formation, such as hamartoma formation in other sites of the body, is unknown. Though previous theories have suggested that because the midline dorsal tongue is a fusion point for different types of embryologic tissue, there may be a predisposition for this location to generate hamartomatous lesions [24]. While these lesions are often present as an isolated finding, they can also be present in the setting of a genetic syndrome, particularly oral-facial-digital syndromes (OFDS) [26].

There does not appear to be a standardized approach to the workup of these lesions. A review of the literature indicates that providers have utilized both CT and MRI for further evaluation; however, the diagnosis is ultimately made histologically [5–27]. Microscopically, lingual leiomyomatous hamartomas demonstrate smooth muscle predominance but may have various tissue types interspersed throughout [1]. Tissue types are typically visible on histological examination using hematoxylin and eosin staining, which is sufficient to diagnose a leiomyomatous hamartoma. However, special stains, such as smooth muscle actin staining in our case, or S-100 staining to differentiate between leiomyomatous hamartoma and leiomyoma, may help to further clarify the diagnosis of leiomyomatous hamartoma if there is uncertainty [28].

As with any midline tongue lesion, it is important to evaluate for the presence of a lingual and native thyroid gland, as premature excision of the lingual mass could induce a hypothyroid state if there is inadequate native thyroidal tissue [29]. The presence or absence of a lingual and native thyroid may be confirmed via ultrasound or higher resolution imaging (e.g., MRI), as was performed in our case [30]. Surgical excision is strongly recommended as morbidity is low and the often-simple procedure is curative, particularly given the non-neoplastic nature of the lesion. To date, there have been no recurrences of lingual hamartomas, particularly those of leiomyomatous composition, reported in the literature. There have been reports of nonlingual head and neck hamartomas recurring, though these recurred due to incomplete excision and only at a rate of approximately 3% [31].

## 4. Conclusion

Hamartomatous lesions, defined by their predominant tissue type, may be present throughout the body and rarely occur in the tongue and tongue base. Our case is one of only 30 total cases reported in the literature since 1945 of lingual leiomyomatous hamartomas. When presented with a midline tongue lesion, hamartomas should be included in the differential with lingual thyroid, various cystic lesions, polyps, macroglossia, and neoplasia. Workup of these lesions should include imaging, preferably with higher resolution (e.g., CT and MRI) and histological examination. A diagnosis of lingual leiomyomatous hamartoma is made histologically. Treatment is excision with wide margins and is curative.

## Figures and Tables

**Table 1:** Summary of lingual leiomyomatous hamartoma cases reported in the English literature to date.

Authors	Year	Number of cases	Age of diagnosis	Sex	Location of lesion (s)	Tissue composition
Stamm and Tauber [5]	1945	1	At birth	F	Tongue base	Smooth muscle, adipose, vessels, salivary glands, and connective
Perri [6]	1956	1	34 years	F	Tongue base	Smooth muscle, salivary glands, and adipose, covered with nonkeratinizing squamous epithelium
Hinshaw [7]	1963	1	4 years	F	Tongue base	Salivary glands, smooth and striated muscle, and lymphatic
Ishii [8]	1968	1	4 months	F	Tongue tip, tongue base, left lateral tongue	Stratified squamous epithelium, collagenous/fibrotic tissue, smooth muscle, salivary glands, and adipose
Demuth and Johns [9]	1981	1	18 months	M	Tongue base	Nerve elements, smooth muscle, skeletal muscle, and glands
Becker et al. [10]	1984	1	At birth	M	Tongue base	Squamous epithelium, smooth muscle, minor salivary glands, nervous, and fibrovascular
Goldsmith et al. [11]	1995	1	1 year and 4 months	M	Dorsal tongue	Fibrous tissue, smooth muscle, thin- and thick-walled vessels, and minor salivary glands
de la Rosa-García and Mosqueda-Taylor [12]	1999	1	6 years	M	Tongue tip	Smooth muscle, fibrous stroma, dilated vascular channels, and nerve fibers
Kobayashi et al. [13]	2001	1	3 months	M	Dorsal tongue	Parakeratinized stratified squamous epithelium, fascicular or aggregated smooth muscle, and striated muscle
Krieger et al. [2]	2007	5	1.8 days	1. M	All dorsal tongue	All containing smooth muscle; 4/5 had variably sized vessels; 1/5 had vessels; and salivary glands
			2.4 months	2. F		
			3.5 months	3. F		
			4.1 year	4. M		
			5.5 years	5. M		

Goold et al. [14]	2007	1	5 months	M	Tongue base	Smooth muscle, squamous epithelium, and mucous glands
Iida et al. [15]	2007	1	2 years and 7 months	M	Midline and posterior dorsal tongue (2 lesions)	Smooth muscle, collagen fibers, nerve fibers, and small vessels
Nava-Villalba et al. [16]	2008	1	5 months	M	Dorsal tongue	Parakeratinized stratified squamous epithelium, nerves, lymphatics, blood vessels, and smooth muscle
de faria et al. [17]	2008	1	61 years	F	Dorsal tongue	Smooth muscle-like spindle cells, collagenous stroma, small- to medium-sized blood vessels with thin or thick walls, adipose, mucinous minor salivary gland tissue, nerve fibers, and small leukocyte aggregates
Kuperan et al. [18]	2012	1	5 months	M	Dorsal tongue	Smooth muscle (other components not specified)
Nakanishi et al. [19]	2012	1	3 years	M	Dorsal tongue	Stratified squamous epithelium, spindle cells within submucoepithelial connective tissue, and fibrous, adipose, and minor salivary gland tissue
Wang et al. [20]	2013	1	29 years	M	Dorsal tongue	Stratified squamous epithelium, smooth muscle, blood vessels, nervous tissue, and adipose tissue in fibrous stroma
Majumder et al. [21]	2014	1	Infant (unspecified age)	M	Dorsal tongue	Squamous mucosa, smooth muscle, dilated lymphatics, salivary glands, and skeletal muscle
Fadzilah et al. [22]	2016	1	6 weeks	M	Tongue base	Stratified squamous epithelium, smooth muscle fibers, occasional adipose tissue, and vascular channels
Nguyen et al. [23]	2018	1	20 years	M	Dorsal tongue	Fibrovascular connective tissue, smooth muscle, skeletal muscle, adipose, salivary tissue, blood vessels, lymphoid tissue, peripheral nerves, and ganglion cells
Sánchez-Romero et al. [24]	2019	3	1.10 years	1. F	1. Dorsal tongue	All 3 contained nonkeratinizing stratified squamous epithelium, smooth muscle, skeletal muscle, connective tissue clefts, small nerve bundles and blood vessels, and adipose tissue. Only case 1 had salivary gland tissue.
			2.15 years	2. F	2. Dorsal tongue	
			3.4 years	3. F	3. Dorsal tongue	

Liu et al. [26]	2020	7	1.3 months	1. F	1. Dorsal tongue	All with haphazard arrangement of smooth muscle within underlying submucosa with interposed minor salivary glands and ducts
			2.5 months	2. F	2. Dorsal and base of tongue	
			3.6 months	3. M	3. Dorsal tongue	
			4.7 months	4. F	4. Dorsal tongue	
			5.9 months	5. M	5. Dorsal tongue	
			6.11 months	6. M	6. Dorsal tongue	
			7.14 months	7. M	7. Dorsal tongue	

Arredondo Montero et al. [25]	2022	1	2 months	F	Dorsal tongue	Squamous epithelium, smooth muscle, adipose tissue, and seromucous glands
